# ATM mutations improve radio-sensitivity in wild-type isocitrate dehydrogenase-associated high-grade glioma: retrospective analysis using next-generation sequencing data

**DOI:** 10.1186/s13014-020-01619-y

**Published:** 2020-07-31

**Authors:** Nalee Kim, Se Hoon Kim, Seok-Gu Kang, Ju Hyung Moon, Jaeho Cho, Chang-Ok Suh, Hong In Yoon, Jong Hee Chang

**Affiliations:** 1 Department of Radiation Oncology, Yonsei Cancer Center, Severance Hospital, Yonsei University Health System, Yonsei University College of Medicine, 50-1 Yonsei-ro, Seodaemun-gu, Seoul, 03722 Republic of Korea; 2 Department of Radiation Oncology, Samsung Medical Center, Sungkyunkwan University School of Medicine, Seoul, Republic of Korea; 3 Department of Pathology, Severance Hospital, Yonsei University Health System, Yonsei University College of Medicine, 50-1 Yonsei-ro, Seodaemun-gu, Seoul, 03722 Republic of Korea; 4 Brain Tumor Center, Severance Hospital, Yonsei University Health System, Yonsei University College of Medicine, 50-1 Yonsei-ro, Seodaemun-gu, Seoul, 03722 Republic of Korea; 5 Department of Neurosurgery, Severance Hospital, Yonsei University Health System, Yonsei University College of Medicine, 50-1 Yonsei-ro, Seodaemun-gu, Seoul, 03722 Republic of Korea; 6 Department of Radiation Oncology, CHA Bundang Medical Center, CHA University, Gyeonggi-Do, Republic of Korea

**Keywords:** ATM, IDH-wild type high-grade glioma, Radiosensitivity, Next-generation sequencing, Radiation therapy

## Abstract

**Background:**

To identify the association between somatic ataxia-telangiectasia mutated (*ATM*) mutations and improved radio-sensitivity, we retrospectively reviewed next-generation sequencing data from patients diagnosed with isocitrate dehydrogenase (IDH)-wildtype high-grade glioma.

**Methods:**

We included 39 individuals with (IDH)-wildtype high-grade glioma (diffuse astrocytoma *n* = 2, anaplastic astrocytoma *n* = 10, and glioblastoma *n* = 27) not subjected to gross tumor resection and undergoing radiation therapy with a median total dose of 60 Gy in 30 fractions. The mutational status of the *ATM* gene was obtained through next-generation sequencing using a TruSight Tumor 170 cancer panel. Disease progression was defined according to the Response Assessment in Neuro-Oncology (RANO) criteria as well as neurologic and clinical findings.

**Results:**

Among the 39 samples, *ATM* mutations (*ATM* mut(+)) were detected in 26% of cases (*n* = 10). No significant differences were observed in the characteristics of the patients or tumors. Among the 10 patients in the *ATM* mut(+) group, there were 6 patients with glioblastoma and 4 patients with anaplastic astrocytoma. Most mutations were missense mutations (*n* = 8, 80%). With a median follow-up of 16.5 mo (interquartile range, 11.4–19.8), *ATM* mut(+) exhibited 1-year in-field control of 100% compared with 44.1% in the *ATM* mut(−) group (*p* = 0.002). There was no difference in the out-field control rate or overall survival between the two groups (*p* = 0.861 and *p* = 0.247, respectively).

**Conclusions:**

Our results demonstrated that *ATM* mutations might be involved in the increased radio-sensitivity with excellent in-field control despite the aggressive nature of IDH-wildtype high-grade glioma. Further studies are necessary to uncover the potential role of *ATM* as a biomarker and candidate therapeutic target in high-grade gliomas.

## Background

Isocitrate dehydrogenase (IDH)-wildtype high-grade glioma (diffuse astrocytoma, anaplastic astrocytoma, and glioblastoma multiforme [GBM]), being an aggressive brain tumor, has been correlated with poor prognosis despite the best trimodality treatment approaches (surgery, chemotherapy, and radiation therapy (RT)). This poor prognosis has been attributed to the intrinsic radio- and chemo-resistance of the tumor [1]. Although there are several clinical and molecular factors known to be prognostic factors for high-grade glioma, including methylation of O-6-methylguanine-DNA methyltransferase (*MGMT*) promoter, age, involvement of the subventricular zone (SVZ), extent of resection, and sex [2], most patients are treated with a uniform adjuvant treatment approach (i.e., one-size-fits-all).

The ataxia-telangiectasia mutated (*ATM*) gene encodes a serine/threonine protein kinase known to be activated by autophosphorylation upon DNA double-strand breaks arising from ionizing radiation. The prevalence of *ATM* mutations (*ATM* mut(+)) in high-grade glioma is known to be less than 5% [3]; hence, owing to this rarity, there have been no clinical reports on such patients. Similar to the approach employing a PARP inhibitor targeting BRCA1 mutations in breast and ovarian cancers [4], several preclinical studies on the inhibition of ATM have been performed to enhance radio-sensitivity in other solid tumors [5, 6] and high-grade gliomas [7–10]. Rainey et al. discovered that transient inhibition of *ATM* by chemicals (CP466722) sensitizes *HeLa* cells and cells expressing *BCR-Abl* to ionizing radiation [6]. Recently, Golding et al. demonstrated that dynamic *ATM* inhibition with sub-micromolar concentrations of KU-60019 slightly increased RT-induced cell killing in human glioblastoma cells [8]. These studies intrigued many physicians to identify the clinical significance of *ATM* mut(+) in real practice.

With the emergence of state-of-the-art sequencing, next-generation sequencing (NGS) [11], physicians have easy access to the *ATM* mutational status of every patient. In this context, we undertook a retrospective analysis of NGS data to address the radio-sensitivity of *ATM* mut(+) and its impact on clinical outcomes in patients with IDH-wildtype high-grade glioma.

## Methods

### Patient selection

As NGS data for gliomas have been utilized in our institution from 2017, patients with newly diagnosed WHO grade II with IDH-wildtype, WHO grade III and IV type gliomas, according to the new 2016 WHO classification, were screened between June 2017 and December 2018. Accordingly, we screened for patients treated with RT following surgery and patients with available NGS data (*n* = 144). Patients were excluded from the study if one of the following criteria was met: (1) they had undergone gross total removal of the tumor, and thus it was not possible to investigate the response of the residual tumor to RT (*n* = 66); (2) they had been previously diagnosed with a primary CNS tumor (*n* = 15); (3) they had tumors with IDH mutations (*n* = 9); (4) they had undergone hypofractionated RT (*n* = 2); (5) peritumoral edema was not included in RT fields (*n* = 5); (6) they could not provide follow-up images (n = 5); and (7) they could not complete RT (*n* = 3). Finally, 39 patients, including 10 patients with *ATM* mutations (*ATM* mut(+) group) and 29 patients without *ATM* mutations (*ATM* mut(−) group), were included in our cohort. The study protocol was approved by the institutional review board (No.4–2019-0009), and the requirement for the provision of informed consent was waived because of the retrospective nature of this study.

### Multi-modal treatments

Treatments and follow-up for every patient were performed by a multidisciplinary neuro-oncology board, including neurosurgeons, radiation oncologists, neuro-radiologists, neuropathologists, and medical oncologists [2]. All patients were evaluated based on perioperative and follow-up magnetic resonance images (MRI) and clinical symptoms. The involvement of the SVZ was assessed via preoperative MRI according to a standardized spatial classification [12]. Navigation-guided surgery following the maximal safe resection protocol was performed for all patients, except for those who underwent stereotactic biopsy. The extent of surgery was evaluated using immediate postoperative gadolinium-enhanced T1-weighted MRI obtained within 48 h after surgery and then categorized as total (absence of visible contrast-enhanced portion), subtotal (at least 90% of the tumor removed), partial (less than 90% of the tumor removed), or biopsy (in case of stereotactic biopsy) [13].

Conventional fractionated RT with a median total dose of 60 Gy (interquartile range, IQR: 60.0–60.0) was applied in 30 fractions to the gross tumor volume. With postoperative MRI, both the resection cavity and residual tumor were included in the gross tumor volume. The clinical target volume was delineated to include the peritumoral edema with a 1- or 1.5-cm margin on T2-weighted fluid-attenuated inversion recovery postoperative MRI, and a median total dose of 49.5 Gy (IQR: 48.0–51.0) in 30 fractions was then applied to the clinical target volume [14, 15]. Following the treatment strategy followed at our institution, there was no difference in the dose prescription of the protocol according to pathology: all patients were treated with intensity-modulated RT using Tomotherapy (Hi-Art TomoTherapy; Accuray, Sunnyvale, CA, USA).

In the case of patients with GBM, during RT, all patients concomitantly underwent a daily administration of temozolomide (75 mg/m^2^ of body surface area per day, 7 days per week, from the first to the last day of RT), followed by the administration of adjuvant temozolomide (150–200 mg/m^2^ for 5 days during each 28-day cycle). Regarding patients with anaplastic astrocytoma and WHO grade II with IDH-wildtype, 6 cycles of adjuvant procarbazine, lomustine, and vincristine (oral lomustine; 110 mg/m^2^ on day 1; oral procarbazine: 60 mg/m^2^ per d from day 8 to day 21; and intravenous vincristine: 1.4 mg/m^2^ on day 8 and 29) were administered every 6 weeks for 9 months.

### Molecular analysis

Representative formalin-fixed, paraffin-embedded tissues were analyzed by targeted NGS using the commercially available TruSight Tumor 170 panel (Illumina, Inc., San Diego, CA, USA). Detailed methods for the procedure of sequencing and data analysis have been previously described [16, 17]. In the obtained NGS data, along with *ATM* mutations, we identified other frequent mutations found in gliomas, such as mutations in the Breast Cancer susceptibility gene1/2 (*BRCA1/2*), phosphatase and tensin homolog (*PTEN*), telomerase reverse transcriptase (*TERT*), and tumor protein p53 (*TP53*) genes. The DNA methylation status of the *MGMT* promoter was also examined [18]. Additionally, we stratified patients based on the consensus guidelines from the Consortium to Inform Molecular and Practical Approaches to CNS Tumor Taxonomy (cIMPACT-NOW) for the identification of gliomas appearing histologically as WHO grade II or III with molecular features of GBM using the following criteria: (1) amplification of epidermal growth factor receptor (*EGFR*) (*n* = 1), (2) combined whole chromosome 7 gain and whole chromosome 10 loss, or (3) mutation of the *TERT* promoter (*n* = 5) [19].

### Follow-up

Follow-up of all patients was performed until death or time of analysis. Most patients underwent MRI 1 month after the planned RT as well as every 3 months for the first 2 years, and every 6 to 12 months thereafter according to institutional policy. Disease progression was defined using the Response Assessment in Neuro-Oncology (RANO) criteria [20] as well as neurologic and clinical findings. All patients suspected of having progressive disease were determined as exhibiting progressive disease after discussion and joint decision made by the multidisciplinary team. Either in-field or out-field failure was defined based on the relationship between the RT field (within the region for 95% of the prescribed dose) and the volume of the recurrence tumor assessed by MRI .

### Statistical analysis

Parsons’ Chi-square or Fisher’s exact test were used to analyze categorical variables, whereas the Mann-Whitney U test (non-normally distributed data) was used to compare continuous variables when comparing differences in the characteristics of patients and treatments between the two groups. All events (including in-field failure, out-field failure, and death) were measured from the day of diagnosis (day of surgery) to the time of the event. The Kaplan-Meier method was performed to estimate the in-field, out-field, and overall survival (OS) rates with the log-rank test used to assess prognostic significance. Univariable analyses of in-field and out-field control as well as OS were performed using Cox regression analysis. Further multivariable analysis was not performed because there was no statistically significant factor identified in the univariable analysis. A *p*-value < 0.05 was considered statistically significant. Statistical analyses were performed using SPSS (version 25.0.0; IBM Corp., Armonk, NY, USA) and R (version 3.6.2; R Foundation for Statistical Computing, Vienna, Austria; https://www.R-project.org/) software.

## Results

The median age at diagnosis was 59 years (IQR: 48–63), and most patients presented with a Karnofsky performance scale (KPS) score ≤ 80. Perioperative MRI revealed that 87.2% of tumors were located in the SVZ, and 48.7% of patients underwent subtotal removal. Methylation of the *MGMT* promoter was observed in 61.5% of patients. There was no significant difference in the characteristics of the patients and treatments between the *ATM* mut(+) and *ATM* mut(−) groups (Table 1). In addition, there was no difference in the frequency of mutations in other genes (Additional file 1). Detailed information on each patient with an *ATM* mutation is listed in Table 2. Missense mutations (8 patients, 80%) were the most common mutations in the *ATM* mut(+) group. There were six patients diagnosed with GBM and four patients with IDH-wildtype anaplastic astrocytoma. Based on preoperative MRI, the involvement of the SVZ (9 patients, 90.0%) and the presence of gliomatosis (6 patients, 60.0%) were frequently observed in the *ATM* mut(+) group.

**Table 1:** Patient and treatment characteristics

	Total	*ATM* mut(−)	*ATM* mut(+)	*p*-value
	*N* = 39	*N* = 29	*N* = 10	
Median age [IQR], years	59.0 [47.5;63.0]	60.0 [52.0;64.0]	49.0 [37.0;59.0]	0.071
< 60 years, *n* (%)	22 (56.4)	14 (48.3)	8 (80.0)	0.169
≥ 60 years, *n* (%)	17 (43.6)	15 (51.7)	2 (20.0)	
Sex, *n* (%)				0.727
Male	20 (51.3)	14 (48.3)	6 (60.0)	
Female	19 (48.7)	15 (51.7)	4 (40.0)	
Preoperative KPS, *n* (%)				0.580
≤ 80	28 (71.8)	22 (75.9)	6 (60.0)	
90–100	11 (28.2)	7 (24.1)	4 (40.0)	
Subventricular zone, *n* (%)				1.000
Free	5 (12.8)	4 (13.8)	1 (10.0)	
Involvement	34 (87.2)	25 (86.2)	9 (90.0)	
Gliomatosis, *n* (%)				0.515
No	21 (53.8)	17 (58.6)	4 (40.0)	
Yes	18 (46.2)	12 (41.4)	6 (60.0)	
Pathology*, *n* (%)				0.380
Diffuse astrocytoma	2 (5.1)	2 (6.9)	0 (0.0)	
Anaplastic astrocytoma	10 (25.7)	6 (20.7)	4 (40.0)	
Glioblastoma	27 (69.2)	21 (72.4)	6 (60.0)	
*MGMT* promoter, *n* (%)				1.000
Unmethylated	15 (38.5)	11 (37.9)	4 (40.0)	
Methylated	24 (61.5)	18 (62.1)	6 (60.0)	
Median Ki67 index [IQR], %	15.0 [6.5;26.2]	15.0 [6.5;27.5]	17.5 [7.5;30.0]	0.617
< 15%, *n* (%)	16 (41.0)	12 (41.4)	4 (40.0)	1.000
≥ 15%, *n* (%)	23 (59.0)	17 (58.6)	6 (60.0)	
Extent of resection, *n* (%)				0.562
Biopsy	2 (5.1)	2 (6.9)	0 (0.0)	
Partial removal	18 (46.2)	14 (48.3)	4 (40.0)	
Subtotal removal	19 (48.7)	13 (44.8)	6 (60.0)	
Median total RT dose [IQR], Gy	60.0 [60.0;60.0]	60.0 [60.0;60.0]	60.0 [60.0;60.0]	0.600
Median total RT fractions [IQR], fx	30.0 [30.0;30.0]	30.0 [30.0;30.0]	30.0 [30.0;30.0]	0.631

*Abbreviations*: *IQR* interquartile range, *ATM* ataxia-telangiectasia mutated gene, *mut* mutation, *KPS* Karnofsky performance status, *MGMT* O[6]-methylguanine-DNA methyltransferase, *RT* radiation therapy, *Gy* gray, *fx* fractions

* Pathology refers to the WHO grade

**Table 2:** Detailed information on patients harboring the *ATM* mutation

Patient number	#1	#2	#3	#4	#5	#6	#7	#8	#9	#10
Mutation	Missense mutation	Missense mutation	Missense mutation	Missense mutation	Missense mutation	Frameshift deletion	Missense mutation	Missense mutation	Frameshift insertion	Missense mutation
VAF (%)	43.01	53.22	46.3	79.92	11.8	25.88	92.49	6.45	5.08	0.369
Amino acid change	p.R2832H	p.R924O	p.K92T	p.P2974L	p.L822S	p.L2946Nfs*9	p.K92T	p.L413I	p.S28212Vfs*3	p.P260T
Sequence change	c.8495G > A	c.2771G > A	c.275A > C	c.8921C > T	c.2465 T > C	c.8835_8836delGT	c.275A > C	c.1237C > A	c.8432dupA	c.778C > A
Age, years	63	51	47	35	32	43	37	58	63	59
Sex	Male	Female	Female	Female	Male	Male	Female	Male	Male	Male
KPS	70	90	80	90	80	80	90	80	100	60
Pathology	GBM	GBM	GBM	GBM	GBM	GBM	AA, IDH-WT	AA, IDH-WT	AA, IDH-WT	AA, IDH-WT
*MGMT* promoter methylation	(−)	(+)	(+)	(−)	(+)	(+)	(−)	(−)	(+)	(+)
SVZ involvement	Yes	None	Yes	Yes	Yes	Yes	Yes	Yes	Yes	Yes
Gliomatosis	No	No	Yes	Yes	No	Yes	No	Yes	Yes	Yes
Extent of resection	Subtotal	Subtotal	Partial	Subtotal	Subtotal	Subtotal	Partial	Subtotal	Partial	Subtotal
Total RT dose, Gy	60	60	60.2	60	60	60	60	60.2	60	60
PD	Yes	Yes	No	Yes	No	No	No	Yes	No	No
PD interval, months	11.6	18.9	18.0	14.4				15.4		
Progression site*	Out-field	Out-field	Out-field	Out-field				Out-field		
Salvage treatment	CTx	Re-RT	BSC	BSC				Re-RT		
Follow-up, months	22.7	19.8	20.8	15.0	8.1	11.4	18.7	17.3	19.2	11.1
Survival	Dead	Alive	Dead	Dead	Alive	Alive	Alive	Alive	Alive	Alive

*Abbreviations*: *VAF* Variant Allele Frequency, *KPS* Karnofsky performance status, *GBM* glioblastoma, *AA* anaplastic astrocytoma, *IDH* isocitrate dehydrogenase, *WT* wild-type, *MGMT* O[6]-methylguanine-DNA methyltransferase, *SVZ* subventricular zone, *RT* radiation therapy, *PD* progressive disease, *CTx* chemotherapy, *Re-RT* re-irradiation, *BSC* best supportive care

* Progression site was defined based on relationship between radiation field and recurrence site

**Fig. 1:**
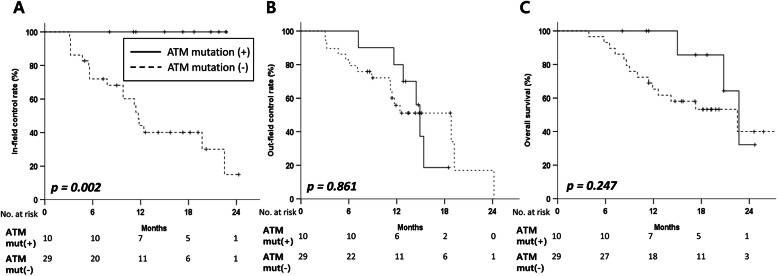
In-field **a** and out-field **b** control rates, and overall survival **c** of patients according to the mutational status of *ATM*

The median follow-up for all patients was 16.5 mo (IQR: 11.4–19.8), and there was no difference in the follow-up period between the two groups (*ATM* mut(+): 18.0 mo (IQR: 11.4–20.8); *ATM* mut(−): 15.5 mo (IQR: 10.0–19.2), *p* = 0.645). Patients with *ATM* mut(+) showed no in-field failure compared with the *ATM* mut(−) group (1-y in-field control rate: 100.0% vs. 44.1%, *p* = 0.002, Fig. 1a). Conversely, there was no difference in the out-field failure between the two groups (1-y out-field control rate: 71.4% vs. 51.9%, *p* = 0.861, Fig. 1b). Of 23 out-field failures, 6 failures (60.0% of *ATM* mut(+)) occurred in the *ATM* mut(+) group and 17 (58.6% of *ATM* mut(−)) in the *ATM* mut(−) group. In addition, out-field failures in the *ATM* mut(+) group were observed to have developed at a median of 15.4 mo (IQR: 11.6–18.9) after diagnosis. In addition, 3 patients died of progressive disease in the *ATM* mut(+) group with a 2-y OS rate of 32.1%, which was comparable to that of the *ATM* mut(−) group (39.9%, *p* = 0.247, Fig. 1c). Using Cox regression univariable analysis, we noted that only the *BRCA* mutation status was associated with worse out-field control (Hazard Ratio, 2.44) and poor OS (Hazard Ratio, 2.78, Table 3).

**Table 3:** Prognostic factors for in-field, out-field control, and overall survival determined using univariable Cox regression analysis

**In-field control**	HR	95% CI	*P*-value
*ATM* (mut(−) vs. mut(+))	0.16	0.00–0.48	0.036
Age at diagnosis (< 60 vs. ≥60)	1.02	0.39–2.69	0.966
Sex (male vs. female)	1.09	0.45–2.78	0.857
KPS (90–100 vs. ≤80)	1.44	0.51–4.07	0.496
SVZ (free vs. involvement)	0.97	0.28–3.39	0.974
Gliomatosis (No vs. Yes)	1.34	0.53–3.39	0.534
Extent of resection (subtotal vs. partial/biopsy)	1.15	0.45–2.94	0.767
Pathology (WHO Grade II-III vs. WHO grade IV)	1.47	0.34–6.43	0.606
* MGMT* promoter (unmethylated vs methylated)	0.59	0.23–1.48	0.260
Ki67 index (< 15% vs. ≥15%)	1.57	0.57–4.32	0.380
*BRCA* status (wild-type vs mutant)	1.46	0.51–4.19	0.485
*PTEN* status (wild-type vs mutant)	1.32	0.47–3.72	0.600
*TERT* status (wild-type vs mutant)	0.93	0.37–2.35	0.873
*EGFR* amplification (No vs. Yes)	1.98	0.74–5.34	0.176
*TP53* status (wild-type vs mutant)	1.39	0.48–4.01	0.546
**Out-field control**	HR	95% CI	P-value
*ATM* (mut(−) vs. mut(+))	0.92	0.35–2.40	0.862
Age at diagnosis (< 60 vs. ≥60)	1.58	0.68–3.71	0.290
Sex (male vs. female)	0.95	0.40–2.26	0.909
KPS (90–100 vs. ≤80)	1.36	0.55–3.39	0.506
SVZ (free vs. involvement)	1.53	0.45–5.22	0.496
Gliomatosis (No vs. Yes)	2.32	0.96–5.61	0.062
Extent of resection (subtotal vs. partial/biopsy)	0.74	0.32–1.74	0.497
Pathology (WHO Grade II-III vs. WHO grade IV)	2.62	0.93–7.38	0.069
*MGMT* promoter (unmethylated vs methylated)	0.49	0.21–1.14	0.098
Ki67 index (< 15% vs. ≥15%)	1.35	0.55–3.33	0.511
*BRCA* status (wild-type vs mutant)	2.44	1.18–6.07	0.035
*PTEN* status (wild-type vs mutant)	0.96	0.35–2.62	0.939
*TERT* status (wild-type vs mutant)	0.48	0.20–1.14	0.096
*EGFR* amplification (No vs. Yes)	3.09	1.09–8.77	0.035
*TP53* status (wild-type vs mutant)	1.80	0.67–4.83	0.246
**Overall survival**	HR	95% CI	P-value
*ATM* (mut(−) vs. mut(+))	0.77	0.27–2.15	0.615
Age at diagnosis (< 60 vs. ≥60)	2.27	0.89–5.84	0.088
Sex (male vs. female)	1.01	0.40–2.54	0.986
KPS (90–100 vs. ≤80)	1.96	0.69–5.57	0.205
SVZ (free vs. involvement)	1.55	0.36–6.79	0.559
Gliomatosis (No vs. Yes)	1.74	0.68–4.46	0.246
Extent of resection (subtotal vs. partial/biopsy)	0.74	0.30–1.84	0.518
Pathology (WHO Grade II-III vs. WHO grade IV)	0.47	0.16–1.43	0.184
*MGMT* promoter (unmethylated vs methylated)	1.04	0.40–2.72	0.931
Ki67 index (< 15% vs. ≥15%)	1.22	0.48–3.10	0.678
*BRCA* status (wild-type vs mutant)	2.78	1.05–7.36	0.039
*PTEN* status (wild-type vs mutant)	1.00	0.33–3.04	0.992
*TERT* status (wild-type vs mutant)	0.88	0.36–2.18	0.785
*EGFR* amplification (No vs. Yes)	1.78	0.67–4.72	0.249
*TP53* status (wild-type vs mutant)	0.42	0.10–1.80	0.241

*The foreparts of the parentheses were set as the reference group

*Abbreviations*: *HR* hazards ratio, *CI* confidence interval, *KPS* Karnofsky performance status, *SVZ* subventricular zone, *MGMT* O[6]-methylguanine-DNA methyltransferase

**Fig. 2:**
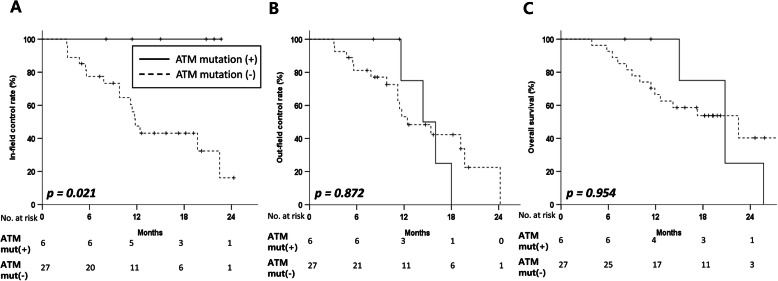
In-field **a** and out-field **b** control rates and overall survival **c** of patients diagnosed with the molecular features of glioblastoma based on cIMPACT-NOW

In subsequent analysis employing the recommended diagnostic criteria for the molecular features of glioblastoma according to cIMPACT-NOW (*n* = 33), *ATM* mut(+) (*n* = 6) was shown to be associated with better in-field control than *ATM* mut(−) (*n* = 27) (1-y in-field control rate: 100.0% vs. 47.5%, *p* = 0.021; Fig. 2a**,** Additional File 2). The out-field control rate and OS values were comparable between the *ATM* mut(+) and *ATM* mut(−) groups in tumors with the molecular features of glioblastoma (Fig. 2b-c**, Additional File****2**). Even in tumors in contact with SVZ, the *ATM* mut(+) group was observed to be significantly associated with better in-field control than *ATM* mut(−) (1-y in-field control rate: 100.0% vs. 48.1%, *p* = 0.003, **Additional File****3**). In addition, there were no differences in the out-field control rate and OS values in tumors involving the SVZ (*p* = 0.711, *p* = 0.433, respectively, **Additional File****3**).

**Fig. 3:**
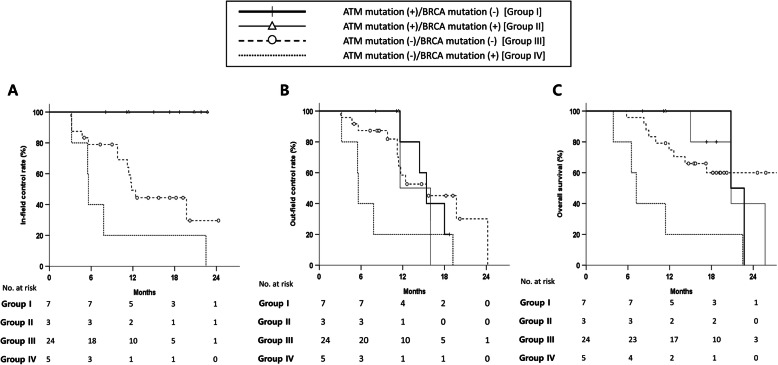
In-field **a** and out-field **b** control rates and overall survival **c** of patients stratified by the mutational status of *ATM* and *BRCA*

As the status of *BRCA* mutations was revealed to function as a prognostic factor for out-field control and OS, we performed further subgroup analysis stratified by the mutational status of *ATM* and *BRCA*. Accordingly, patients with *ATM* mut(−) and *BRCA* mut(+) (*n* = 5) showed the worst clinical outcomes, with a 1-y in-field control rate of 20.0%, out-field control rate of 20.0%, and OS of 20.0% (Fig. 3a-c). In contrast, patients with *ATM* mut(+) and *BRCA* mut(−) (*n* = 7) were observed to exhibit statistically better clinical outcomes, with a 1-y in-field control rate of 100.0% and 2-y OS rate of 40.0% (*p* = 0.002, and *p* = 0.031, respectively). Patients with *ATM* mut(+) and *BRCA* mut(+) (*n* = 3) showed comparable results to the *ATM* mut(+) and *BRCA* mut(−) group (1-y in-field control rate and 1-y OS rate of 100.0%; *p* = 0.809 for OS).

## Discussion

We investigated the clinical impact of *ATM* mutations in patients with intracranial IDH-wildtype high-grade glioma undergoing RT following incomplete tumor resection. Interestingly, although well-known poor prognostic factors, such as either the presence of gliomatosis or the involvement of the SVZ, were predominant in the *ATM* mut(+) group, we observed excellent in-field control without in-field failure. However, it was shown that the *ATM* mut(+) group had no impact on the out-field area, demonstrating comparable out-field failures to the *ATM* mut(−) group. Hence, these clinical results illustrated the increased radio-sensitivity of tumors harboring somatic *ATM* mutations.

There has been considerable accumulated preclinical evidence that *ATM* might play a key role in the response to ionizing radiation. Preclinical studies on cell lines from patients with ataxia-telangiectasia syndromes have demonstrated that these cells exhibit increased sensitivity to radiation [21, 22]. Similarly, experimental inhibition of *ATM* was reported to influence cellular hypersensitivity to radiation [5, 6]. This salutary effect on the radio-sensitivity of cells following the inhibition of *ATM* was recently observed in GBM cell lines [7, 8], xenograft models of GBM [9], and even patient-derived xenograft models [10]. However, there have been few clinical reports agreeing that *ATM* mut(+) could be a prognostic biomarker for a favorable response to RT. Jennifer Ma et al. [23] reported that 8 patients with extracranial primary disease (nonglial tumors) harboring *ATM* mut(+) demonstrated excellent and durable RT responses with a median local control of 4.62 years; moreover, only 2 patients exhibited local recurrence. Especially, 2 patients with breast and non-small cell lung cancers receiving whole brain RT were observed to relapse at 41.0 and 43.2 months after RT. Additionally, Su et al. [24] reported the significant benefit of adjuvant RT in 43 carriers of germline *ATM* mutations diagnosed with breast cancer; they reported that 13 patients treated with RT (not cobalt therapy) remained locally disease-free, sustaining tolerable toxicities. Recently, we reported that somatic *ATM* mutations in solid tumors (except brain tumors) were related to markedly improved responses after RT compared with tumors not harboring *ATM* mutations: an overall response rate of 61.0% (vs. 24.0% in *ATM* mut(−) tumors) with a durable response for a median period of 11 mo (vs. 3 mo in *ATM* mut(−)) [25]. In the current study, the *ATM* mut(+) group was shown to yield excellent in-field control, indicating the radio-sensitive nature of *ATM* mutations even in patients with intracranial high-grade glioma.

Following the generation of radiation-induced double-strand breaks in the DNA, the *ATM* gene is known to control the key signaling pathway in the repair process of the DNA double-strand break damage [3, 26]. After it has been recruited to the site of the double-strand break by the MRE11-RAD50-NBS1 complex, *ATM* is activated by autophosphorylation at Ser1981 [27]. Catalytic activation of *ATM* is known to subsequently lead to the phosphorylation of many downstream effectors involved in the activation of the G1/S cell-cycle checkpoint arrest (CHk1 and Chk2) [28], homologous recombination or non-homologous end-joining repair of DNA [29, 30], and apoptosis (p53) [31]. Consequently, mutations in *ATM* might prevent the repair or restoration of radiation-induced damaged DNA, resulting in the radio-sensitivity of cells. Therefore, germline *ATM* mut(+) is highly involved in radiation-induced risks from its radio-sensitivity. Recently, there is an effort to predict individual response to radiation based on *ATM* nucleoshuttling rate [32]. Future investigations to increase the understanding of *ATM* is needed for refinement and development models of personalized ionizing radiation response.

From a clinical point of view, targeting *ATM* has intrigued many researchers and encouraged them to attempt approaches to enhance the radio-sensitivity of cells. Since then, several *ATM* inhibitors have been developed and their in vitro or in vivo radio-sensitivity have been reported [17]. However, to date, no *ATM* inhibitor has yet been clinically used because of the lack of bioavailability, concerns of potential side-effects, and the lack of clinical trials. There is an ongoing prospective phase I trial on the combination of palliative RT and an *ATM* inhibitor against solid tumors (NCT03225105). In addition to the administration of *ATM* inhibitors as radio-sensitizers, somatic *ATM* mutations could also be applied as intrinsic radio-sensitizers. The advent of NGS in routine clinical practice might allow *ATM* to be used as the sole biomarker for patients treated with RT. Recently, researchers developed a genome-adjusted radiation dose model based on an individualized radio-sensitivity index with genomic features [33]. Beyond personalized radiation dose planning, we could cautiously assume that whole brain or large field RT rather than focal RT might be an effective personalized treatment option with excellent in-field control and prolonged survival in patients with IDH-wildtype *ATM* mut(+) high-grade glioma.

In addition, the current study showed that *BRCA* mutations could be associated with poor outcomes of not only intracranial control but also OS. Our results appeared to differ to some extent from those demonstrated on different primary tumors, many of which have reported the survival benefit of *BRCA* mutations. To date, there has not been any clinical evidence supporting the notion that a somatic *BRCA* mutation might govern the prognosis of IDH-wildtype high-grade glioma. Recent studies of germline *BRCA1/2* mutations in patients with breast cancer have suggested that these mutations have a similar prognosis, though they exhibit different effects on the efficacy of chemotherapy [34, 35]. Additionally, there have been several reports analyzing the survival benefit of *BRCA1/2* mutations in patients with ovarian cancer [36]. These data have collectively supported the relatively better short-term prognosis in patients carrying *BRCA1/2* mutations. In the current study, a subgroup analysis stratified by the mutational status of both the *BRCA* and *ATM* genes revealed that patients without any mutation showed comparable outcomes to the historical data, whereas the *ATM* mut(−)/*BRCA* mut(+) group showed the worst outcomes. The *ATM* mut(+) group with or without *BRCA* mutations showed comparable results of rates of out-field control and OS, with the same excellent in-field control. These results reflected the fact that *BRCA* might be a target in the downstream signaling of *ATM*. Further investigation regarding the prognostic impact of *BRCA* on high-grade glioma is needed.

Our study had several limitations. First, this was a retrospective analysis; thus, the results should be cautiously reviewed. Second, owing to the limited number of patients, there are issues regarding the statistical insignificance of the results and the unavailability of multivariable analysis to minimize potential confounders. However, to our knowledge, this study has the largest clinical data, to date, on tumors with somatic *ATM* mutations analyzed by the latest NGS technology. In addition, a relatively frequent *ATM* mut(+) in the current cohort compared to previous report could overestimate the potential predictive value of *ATM* mut(+); however, the widespread utilization of NGS is expected to clarify the real-world prevalence of *ATM* mut(+). An additional limitation was that this was a single-center study; however, its consistent treatment strategy of surgery and RT provides another strength to this study. Lastly, the in-field control rates of the *ATM* mut(+) group could have been overestimated owing to the short-term follow-up of this study. As most patients with IDH-wildtype high-grade glioma were observed to experience in-field or out-field failures within 1 year after diagnosis, the current follow-up period was considered acceptably reasonable for the analysis of failure patterns. However, long-term follow-up data based on a larger number of patients is still needed to efficiently determine certain effects of *ATM* mutations.

## Conclusions

We demonstrated that *ATM* mutations in IDH-wildtype high-grade glioma could yield an excellent RT response, irrespective of the adverse features of gliomatosis or the involvement of the SVZ. Owing to the limited number of *ATM* mutations in high-grade glioma, a further validation in a larger cohort, even including patients under gross total removal status, is needed to identify the prognostic value of *ATM* mutations. Therefore, we have initiated a multicenter cohort study with Korean Radiation Oncology Group (Protocol No. KROG 19–11). Further preclinical and prospective clinical studies are warranted to elucidate the role of large field RT in patients with *ATM* mut(+) and the effect of the combination of RT and *ATM* inhibitors during the course of RT in IDH-wildtype high-grade glioma.

## Supplementary information

**Additional file 1. **Mutations in other genes according to the mutational status of *ATM.)*

**Additional file 2. **Clinical outcomes according to the mutational status of *ATM* in tumors with the molecular features of glioblastoma based on cIMPACT-NOW.

**Additional file 3.** In-field (a) and out-field (b) control rates and overall survival (c) of patients presenting SVZ involvement.

## Data Availability

It is limited due to institutional data protection law and confidentiality of patient data.

## Abbreviations

IDH: Isocitrate dehydrogenase
RT: Radiation therapy
MGMT: O-6-methylguanine-DNA methyltransferase
SVZ: Subventricular zone
ATM: Ataxia-telangiectasia mutated
NGS: Next-generation sequencing
IQR: Interquartile range
MRI: Magnetic resonance image
cIMPACT-NOW: Consortium to Inform Molecular and Practical Approaches to CNS Tumor Taxonomy
BRCA1/2: Breast Cancer susceptibility gene1/2
OS: Overall survival
KPS: Karnofsky performance scale
GBM: Glioblastoma multiforme
