# Supplementary material for: ATM mutations improve radio-sensitivity in wild-type isocitrate dehydrogenase-associated high-grade glioma: retrospective analysis using next-generation sequencing data
Source: Radiat Oncol. 2020 Jul 31;15:184. doi: 10.1186/s13014-020-01619-y (PMC7393839; doi:10.1186/s13014-020-01619-y)
Supplement: Supplementary file 1 — Additional file 1. Mutations in other genes according to the mutational status of ATM.) [file 13014_2020_1619_MOESM1_ESM.docx]

**Additional file 1. Mutations in other genes according to the mutational status of *ATM***

|  | Total | *ATM* mut(-) | *ATM* mut(+) | p-value |
| --- | --- | --- | --- | --- |
|  | n = 39 | n = 29 | n = 10 |  |
|  | n (%) | n (%) | n (%) |  |
| *BRCA** | 8 (20.5) | 5 (17.2) | 3 (30.0) | 0.684 |
| *PTEN* | 10 (25.6) | 8 (27.6) | 2 (20.0) | 0.957 |
| *TERT promoter* | 22 (56.4) | 18 (62.1) | 4 (40.0) | 0.399 |
| *EGFR amplification* | 9 (23.1) | 8 (27.6) | 1 (10.0) | 0.482 |
| *TP53* | 9 (23.1) | 7 (24.1) | 2 (20.0) | 1.000 |

*Includes both BRCA1 and BRCA2 mutation
