# Supplementary material for: ATM mutations improve radio-sensitivity in wild-type isocitrate dehydrogenase-associated high-grade glioma: retrospective analysis using next-generation sequencing data
Source: Radiat Oncol. 2020 Jul 31;15:184. doi: 10.1186/s13014-020-01619-y (PMC7393839; doi:10.1186/s13014-020-01619-y)
Supplement: Supplementary file 2 — Additional file 2. Clinical outcomes according to the mutational status of ATM in tumors with the molecular features of glioblastoma based on cIMPACT-NOW. [file 13014_2020_1619_MOESM2_ESM.docx]

**Additional file 2. Clinical outcomes according to the mutational status of *ATM* in tumors with the molecular features of glioblastoma based on cIMPACT-NOW**

|  | **In-field control rate (%)** | | | **Out-field control rate (%)** | | | **Overall survival (%)** | | |
| --- | --- | --- | --- | --- | --- | --- | --- | --- | --- |
|  | *ATM* mut(-) | *ATM* mut (+) | p-value | *ATM* mut(-) | *ATM* mut (+) | p-value | *ATM* mut(-) | *ATM* mut (+) | p-value |
|  | N=27 | N=6 |  | N=27 | N=6 |  | N=27 | N=6 |  |
| 6-month | 77.4 | 100.0 | 0.021 | 81.2 | 100.0 | 0.872 | 92.6 | 100.0 | 0.954 |
| 1-year | 47.5 | 100.0 |  | 53.2 | 75.0 |  | 66.5 | 100.0 |  |
| 2-year | 16.2 | 100.0 |  | 33.9 | 0.0 |  | 40.3 | 25.0 |  |
