# Supplementary material for: ATM mutations improve radio-sensitivity in wild-type isocitrate dehydrogenase-associated high-grade glioma: retrospective analysis using next-generation sequencing data
Source: Radiat Oncol. 2020 Jul 31;15:184. doi: 10.1186/s13014-020-01619-y (PMC7393839; doi:10.1186/s13014-020-01619-y)

**Additional file 3. In-field (a) and out-field (b) control rates and overall survival (c) of patients presenting SVZ involvement**


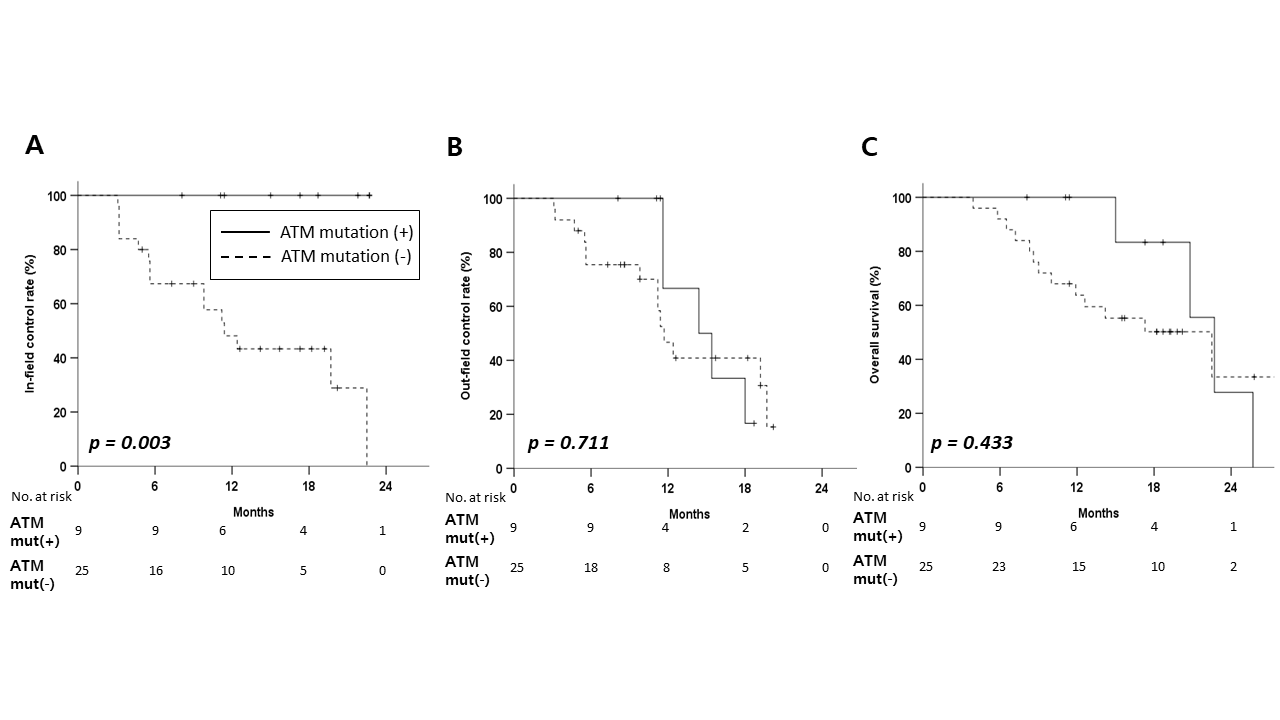

Supplement: Supplementary file 3 — Additional file 3. In-field (a) and out-field (b) control rates and overall survival (c) of patients presenting SVZ involvement. [file 13014_2020_1619_MOESM3_ESM.docx]
